# RNA Sequencing Data Sets Identifying Differentially Expressed Transcripts during Campylobacter jejuni Biofilm Formation

**DOI:** 10.1128/MRA.00982-19

**Published:** 2020-01-02

**Authors:** Greg Tram, William P. Klare, Joel A. Cain, Basem Mourad, Stuart J. Cordwell, Victoria Korolik, Christopher J. Day

**Affiliations:** a Institute for Glycomics, Griffith University, Southport, Queensland, Australia; b Charles Perkins Centre, The University of Sydney, Sydney, New South Wales, Australia; c School of Life and Environmental Sciences, The University of Sydney, Sydney, New South Wales, Australia; Queens College

## Abstract

Campylobacter jejuni is a foodborne pathogen and an important contributor to gastroenteritis in humans. C. jejuni readily forms biofilms which may play a role in the transmission of the pathogen from animals to humans. Herein, we present RNA sequencing data investigating differential gene expression in biofilm and planktonic C. jejuni. These data provide insight into pathways which may be important to biofilm formation in this organism.

## ANNOUNCEMENT

Campylobacter jejuni is responsible for a large proportion of bacterial gastroenteritis infections in developed nations and is a precursor to the onset of neuroparalytic conditions such as Guillain-Barré and Fisher syndromes (1–3). Biofilms have been suggested to play a role in the transmission of this pathogen from animals and birds, where it is part of the normal intestinal flora, to humans, enabling the organism to remain infectious under harsh environmental conditions (4–6). Using RNA isolated from both biofilm-encased and planktonic cells, we demonstrated that there is a high level of change in the C. jejuni transcriptome during biofilm formation.

The wild-type C. jejuni strain 11168-O, used in this study, was provided by Diane Newell (7). Strains were initially grown microaerobically overnight at 42°C on Columbia blood agar with Skirrow supplement. Planktonic cell samples were prepared by inoculating heart infusion broth with overnight growth (Oxoid, United Kingdom) for a period of 12 hours under microaerobic conditions at 42°C with shaking. Biofilms were prepared by inoculating Mueller-Hinton broth with an overnight growth (Oxoid, United Kingdom) and allowing biofilm to form statically on the surface in glass petri dishes under aerobic conditions at 42°C. Cells were then harvested and RNA was prepared using modified CsCl density gradient ultracentrifugation as described previously (8).

RNA library construction was carried out using an Epicentre ScriptSeq library preparation kit v2 with 13 cycles of library amplification. Sequencing was performed by the Micromon RNA sequencing facility at Monash University using an Illumina NextSeq 500 instrument with a run configuration setting of midoutput 75PE (paired end). Quality control (QC) of the raw transcriptome sequencing (RNA-seq) reads was performed at Illumina BaseSpace using the FastQ toolkit v2.0. Raw reads were trimmed of Illumina TruSeq sequencing adapters and filtered for an average Phred Q score minimum of >30 and a minimum read length of 32 nucleotides (nt) after trimming. Reads were further processed by trimming the 3′ ends of bases with a Q score of <30. Processed output files were aligned to the C. jejuni NCTC 11168 genome (GenBank assembly number GCA_000009085) using Bowtie2 v2.2.5. Output SAM files were converted to binary alignment map (BAM) format using SAMtools v0.1.19 and name sorted prior to input into HTSeq v0.6.1. HTSeq counting was performed in union mode. Default parameters were used for all QC, alignment, and feature-counting operations listed above. Differential gene expression analysis was performed using the edgeR R package. Low-expression reads were filtered from the analysis, and a minimum false discovery rate (FDR) of <0.01 was accepted as differentially expressed after Benjamini-Hochberg *post hoc* correction.

RNA-seq analysis identified 1,571 genes equating ∼96.8% coverage of the C. jejuni NCTC 11168 genome. A total of 789 genes were considered differentially expressed using a fold change cutoff of log_2_ less than –1 and greater than 1 (equivalent to a ±2-fold change) with 427 upregulated and 362 downregulated transcripts in biofilm conditions.

### Data availability.

The data are accessible through the NCBI Gene Expression Omnibus (GEO) Series accession number GSE133783.
